# Research on early warning of renal damage in hypertensive patients based on the stacking strategy

**DOI:** 10.1186/s12911-022-01889-4

**Published:** 2022-08-09

**Authors:** Qiubo Bi, Zemin Kuang, E. Haihong, Meina Song, Ling Tan, Xinying Tang, Xing Liu

**Affiliations:** 1 School of Computer Science, Beijing University of Posts and Telecommunications, Beijing, 100876 China; 2 Department of Hypertension, Beijing Anzhen Hospital of Capital Medical University, Beijing, 100029 China; 3 Department of Cardiology, The First People’s Hospital of Chenzhou, The University of South China, Chenzhou, 423000 China; 4 Department of Anesthesiology, Third Xiangya Hospital, Central South University, Changsha, 410013 China

**Keywords:** Hypertension, Renal damage, Risk assessment, Data mining, Feature engineering, Stacking model

## Abstract

**Background:**

Among the problems caused by hypertension, early renal damage is often ignored. It can not be diagnosed until the condition is severe and irreversible damage occurs. So we decided to screen and explore related risk factors for hypertensive patients with early renal damage and establish the early-warning model of renal damage based on the data-mining method to achieve an early diagnosis for hypertensive patients with renal damage.

**Methods:**

With the aid of an electronic information management system for hypertensive out-patients, we collected 513 cases of original, untreated hypertensive patients. We recorded their demographic data, ambulatory blood pressure parameters, blood routine index, and blood biochemical index to establish the clinical database. Then we screen risk factors for early renal damage through feature engineering and use Random Forest, Extra-Trees, and XGBoost to build an early-warning model, respectively. Finally, we build a new model by model fusion based on the Stacking strategy. We use cross-validation to evaluate the stability and reliability of each model to determine the best risk assessment model.

**Results:**

According to the degree of importance, the descending order of features selected by feature engineering is the drop rate of systolic blood pressure at night, the red blood cell distribution width, blood pressure circadian rhythm, the average diastolic blood pressure at daytime, body surface area, smoking, age, and HDL. The average precision of the two-dimensional fusion model with full features based on the Stacking strategy is 0.89685, and selected features are 0.93824, which is greatly improved.

**Conclusions:**

Through feature engineering and risk factor analysis, we select the drop rate of systolic blood pressure at night, the red blood cell distribution width, blood pressure circadian rhythm, and the average diastolic blood pressure at daytime as early-warning factors of early renal damage in patients with hypertension. On this basis, the two-dimensional fusion model based on the Stacking strategy has a better effect than the single model, which can be used for risk assessment of early renal damage in hypertensive patients.

## Background

According to the 2020 international society of hypertension global hypertension practice guidelines, hypertension is related to cerebrovascular disease and ischemic heart disease. It is also a major risk factor for the incidence and death due to chronic kidney disease [1]. Hypertension can affect the function of organs in the whole body, and the kidney is most easily affected.

In China, the number of uremic patients caused by hypertension reaches 1.5 million every year [2]. Furthermore, among the problems caused by hypertension, early renal damage is often ignored because of unclear symptoms. The typical symptoms and signs of chronic renal failure gradually appear as time goes by. It can not be diagnosed until the condition is severe and irreversible damage occurs. Identifying such patients early and making correct interventions is a critical challenge for clinicians because it is related to delaying the progress of renal damage and reducing medical expenses and is closely related to the prognosis of patients. Therefore, we need to pay great attention to the early renal damage in hypertensive patients.

In clinical practice, it is hard to realize the early diagnosis of the high-risk population of hypertensive renal damage and guide different patients to choose the most suitable scheme to receive treatment in time. Because of few or no symptoms in the early stage of chronic kidney disease (CKD), most patients with renal damage fail to get a timely diagnosis. Many hypertensive patients who look healthy may have developed CKD, and current methods fail to diagnose these patients fully. We intend to establish an early-warning model based on data mining to evaluate the risk of early renal damage by integrating the relevant factors. The factors including cardiovascular risk factors, blood pressure parameters, biochemical blood indicators, and related biomarkers [3–5]. Then we can use the model to identify the high-risk patients early to make a definite diagnosis and give timely treatment. Then we should explore an effective management mode of early hypertensive renal damage to control the risk factors of this population and reduce the incidence rate and harm of CKD.

## Methods

**Fig. 1:**
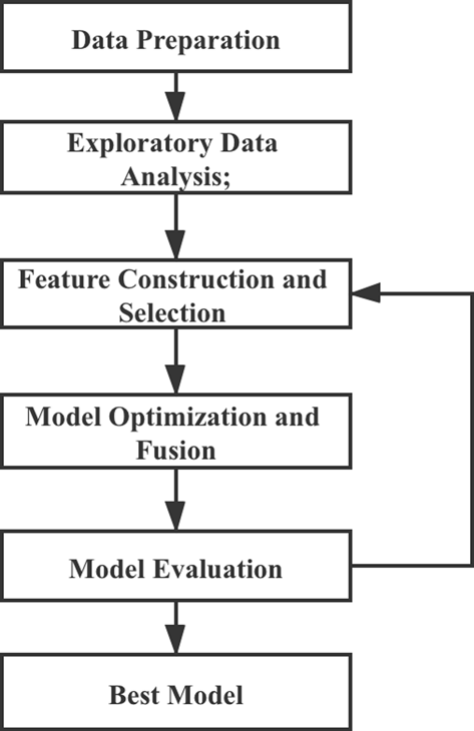
The steps of model construction

In order to achieve the early diagnosis of a high- risk population with hypertensive renal damage, we will screen the early-warning risk factors of early renal damage by feature engineering [6]. Based on these risk factors, we use a data mining approach to establish an early-warning model of renal damage, which fuses three machine learning sub-models: XGBoost, Random Forest, and Extra-Trees by Stacking strategy [7–9]. The specific steps are as follows. (1) data preparation; (2) exploratory data analysis; (3) feature construction; (4) feature selection; (5) model optimization and fusion; (6) model evaluation. The specific process of model construction is shown in Fig. 1.

### Data preparation

From November 2011 to May 2013, Beijing Anzhen Hospital of Capital Medical University (Beijing Institute of Heart Lung and Blood Vessel Diseases), Third Xiangya Hospital of Central South University (Hunan Hypertension Research Center), and Chenzhou No.1 People’s Hospital of Hunan Province (Translational Medicine Institute of University of South China) received 513 patients without complications who have initially diagnosed hypertension. They aged between 35 and 64, including 319 males and 194 females. None of the patients had ever taken any antihypertensive drugs before their visit. According to their albumin- to-creatinine ratio(ACR) levels, the patients are divided into two groups: positive group (30-300mg/g), which is the early renal damage group, and control group (< 30mg/g), which is the normal renal function group. The number of patients in the two groups is 191 and 322, respectively.

In the comparison of the data of the two groups of patients, the levels of fasting blood glucose(FBG), triglyceride(TG), uric acid(UA), and red cell distribution width(RDW) in the positive group are greatly higher than those in the control group. Furthermore, the differences between the two groups are statistically significant (*P* <0.05). The levels of sex ratio, body mass index(BMI), high-density lipoprotein(HDL), low-density lipoprotein(LDL), blood urea nitrogen(BUN), and serum creatinine(Scr) are similar. The differences are not statistically significant (*P* > 0.05). See Table 1.

**Table 1:** The Comparison of clinical and biochemical data

Variable	Control group	Positive group	*P* value
Age	46.37 ± 7.54	47.61 ± 8.14	0.083
Female ratio	36%	40.8%	0.277
BMI (kg/m^2^)	26.03 ± 3.50	25.63 ± 3.26	0.187
HDL (mmol/L)	1.13 ± 0.22	1.10 ± 0.21	0.096
LDL (mmol/L)	3.13 ± 0.87	3.25 ± 0.77	0.136
BUN (mmol/L)	4.80 ± 1.17	4.87 ± 1.07	0.504
Scr (µmol/L)	70.19 ± 13.13	69.63 ± 12.91	0.641
**FBG (mmol/L)**	**5.63 ± 0.67**	**5.76** ± **0.64**	**0.027**
**TG (mmol/L)**	**1.45–2.12**	**1.52–2.34**	**0.047**
**UA (µmol/L)**	**337.99 ± 46.87**	**351.50 ± 49.66**	**0.002**
**RDW (%)**	**12.39 ± 0.63**	**13.32 ± 0.85**	** < 0.001**
ACR (mg/g)	**15.87 ± 8.72**	**74.88 ± 56.42**	** < 0.001**

Bold indicates that the levels of FBG, TG, UA and RDW in the early renal injury group are significantly higher than those in the normal renal function group, and the difference between the two groups is statistically significant (*p* < 0.05)

### Exploratory data analysis

Exploratory data analysis is a data analysis method [10, 11] to explore data structures utilizing mapping, tabulation, equation fitting, calculation of characteristic quantity, and other means for existing data under the minimum prior assumption, specifically including statistical characteristics of data fields, missing situation, distribution, correlation and so on, to facilitate the later feature engineering and model construction.

We conducted exploratory data analysis on the collected hypertension patient data. First, we count the number, missing values, mean, standard deviation, median, minimum, maximum, 25% quantile, 50% quantile, and 75% quantile of individual attributes. Then according to the statistical results, we select the appropriate attributes for the distribution statistics. Finally, we count the P-value of a single attribute, ACR and the correlation coefficient between multiple attributes. The relevant processing results are shown in Tables 2, 3, 4, and 5.

**Table 2:** Missing value statistics

Variable	N	Missing value	%
Hcy	190	323	63.0
2hPBG	219	294	57.3
RVD	313	200	39.0
RVOT	317	196	38.2
WBC	322	191	37.2
RBC	323	190	37.0
Hb	323	190	37.0
Plt	323	190	37.0
A_peak_max	325	188	36.6
EA	325	188	36.6
IVST	330	183	35.7
LVDS	330	183	35.7
LVM	330	183	35.7
LyVII	330	183	35.7
LVH	330	183	35.7
FS	330	183	35.7
E_peak_max	330	183	35.7
LVPWT	331	182	35.5
EF	331	182	35.5
LVEDD	332	181	35.3
SBP_cv_24 h	396	117	22.8
DBP_cv_24 h	396	117	22.8
Ald	413	100	19.5
PRA	415	98	19.1
Ang2	415	98	19.1
hs-CRP	481	32	6.2
ALT	493	20	3.9
AST	494	19	3.7

**Table 3:** Data distribution statistics

Variable	Distribution	Frequency	Percentage %
ACR	0	322	62.8
	1	191	37.2
BMI	24–28	235	45.8
	< 24	149	29.0
	> 28	129	25.1
Blood pressure type	Dipper	246	48.0
	Non-dipper	231	45.0
	Reverse-dipper	27	5.3
	Deep-dipper	9	1.8
HFBG	No	369	71.9
	Yes	144	28.1
HTG	No	301	58.7
	Yes	212	41.3
LDL-C	No	410	79.9
	Yes	103	20.1
Proteinuria	No	322	62.8
	Yes	191	37.2
Sex	Male	319	62.2
	Female	194	37.8
Age	35–44	225	43.9
	45–54	182	35.5
	55–64	106	20.7
RDW	< 12.2	129	25.1
	12.2–12.7	146	28.5
	12.7–13.2	114	22.2
	> 13.2	124	24.2

**Table 4:** Multi-collinearity analysis

Variable	Tolerance	VIF
cDBP	0.333	3.007
**24hDBP**	**0.010**	**96.394**
**Day DBP**	**0.004**	**262.553**
**Night SBP drop rate**	**0.006**	**176.494**
**Night DBP drop rate**	**0.007**	**150.070**
**NightDBP**	**0.003**	**353.446**
SBP_cv_24 h	0.581	1.720
DBP_cv_24 h	396	1.762
cPP	0.311	3.212
**24hPP**	**0.023**	**42.607**
**Day PP**	**0.005**	**213.029**
**Night PP**	**0.004**	**226.067**

Bold means that the VIF value is greater than 5, indicating that there is multicollinearity

**Table 5:** Data statistical analysis

Variable	Average	Standard deviation	Median	Min	Max	25% Quantile	50% Quantile	75% Quantile
ACR	37.84	2.00	24.90	0.00	295.70	12.80	24.90	36.55
Age	46.83	0.34	46.00	35.00	64.00	40.00	46.00	53.00
BMI	25.88	0.15	25.56	17.48	40.32	23.53	25.56	28.08
Height	168.40	0.38	170.00	145.00	192.00	161.00	170.00	175.00
WT	73.70	0.57	73.00	42.00	138.00	65.00	73.00	81.00
ald	0.16	0.00	0.16	0.01	0.35	0.13	0.16	0.19
Ang2	75.33	1.43	66.92	27.63	229.29	57.89	66.92	84.85
ALT	30.96	1.11	23.00	3.00	222.00	16.00	23.00	36.00
AST	25.35	0.49	22.00	10.00	107.00	19.00	22.00	28.00
BUN	4.82	0.05	4.70	0.84	9.90	4.00	4.70	5.60
Scr	69.98	0.58	71.00	42.00	97.00	59.00	71.00	80.00
TC	5.14	0.04	5.13	3.44	7.89	4.51	5.13	5.72
cPP	52.55	0.48	51.00	25.00	92.00	45.00	51.00	59.00
hs-CRP	2.40	0.22	0.98	0.05	52.15	0.44	0.98	2.17
24hDBP	87.80	0.39	87.00	64.00	122.00	82.00	87.00	93.00
cDBP	99.93	0.39	100.00	69.00	130.00	93.00	100.00	105.50
cv_24 h	11.23	0.14	11.00	1.23	20.27	9.33	11.00	13.00
DBP	91.26	0.40	91.00	64.00	125.00	85.00	91.00	97.00
NightDBP	80.75	0.43	80.00	58.00	118.00	74.00	80.00	86.00
Night SBP drop	11.45	0.30	11.83	-10.00	30.43	6.82	11.83	16.34
*Rate*								
day PP	48.86	0.40	48.00	29.00	81.00	42.00	48.00	54.00
NightSBP	126.86	0.56	126.00	97.00	171.00	118.00	126.00	134.00
Night DBP drop	9.44	0.25	9.87	-9.09	25.76	5.93	9.87	13.36
*Rate*								
Night PP	46.10	0.40	45.00	27.00	81.00	39.50	45.00	52.00
eGFR	105.02	1.09	102.06	61.63	268.03	88.72	102.06	116.34
FS%	37.51	0.43	37.00	23.00	107.00	33.00	37.00	40.00
FBG	5.68	0.03	5.54	3.75	8.78	5.24	5.54	5.99
Hb	150.01	0.88	151.00	85.00	195.00	140.00	151.00	161.00
Clinic heart rate	78.44	0.41	80.00	52.00	104.00	72.00	80.00	84.00
Day avg heart rate	78.44	0.39	78.00	50.00	109.00	73.00	78.00	84.00
Night avg heart rate	65.08	0.35	65.00	42.00	101.00	60.00	65.00	70.00
24hSBP	135.68	0.49	134.00	111.00	175.00	128.00	134.00	141.50
cSBP	152.47	0.53	150.00	123.00	193.00	143.00	150.00	159.00
24hCV	12.29	0.19	11.63	5.00	26.58	9.49	11.63	14.48
hPP_24 h	47.88	0.39	46.00	19.00	79.00	42.00	46.00	53.00
24 h avg heart rate	74.28	0.37	74.00	48.00	106.00	69.00	74.00	79.00
DaySBP	140.12	0.49	139.00	113.00	180.00	133.00	139.00	146.00
LVH	0.47	0.03	0.00	0.00	1.00	0.00	0.00	1.00
LVESD	29.77	0.22	30.00	2.00	44.00	27.00	30.00	32.00
LVEDD	47.29	0.26	47.00	9.70	65.00	45.00	47.00	50.00
LVPWT	9.63	0.10	9.60	6.80	30.00	9.00	9.60	10.00
LVMI	110.57	1.33	108.62	54.41	266.19	94.22	108.62	123.41
RVED	20.30	0.17	20.00	12.00	33.00	18.00	20.00	22.00
RVOTD	28.14	0.36	28.00	16.00	73.00	25.00	28.00	30.00
A_peak_max	75.12	1.09	73.00	26.00	159.00	60.00	73.00	88.00
E_peak_max	79.04	1.13	77.00	30.00	143.00	65.75	77.00	92.00
E/A	1.12	0.02	1.15	0.50	3.39	0.77	1.15	1.36
EF%	65.82	0.34	66.00	33.00	79.00	62.00	66.00	70.00
2hPBG	7.49	0.15	6.99	3.73	16.30	5.90	6.99	8.20
TG	1.85	0.02	1.79	1.00	3.89	1.46	1.79	2.22
LDL-C	3.18	0.04	3.20	1.17	5.92	2.62	73.20	3.74
HDL	1.12	0.01	1.10	0.56	1.91	0.96	1.10	1.26
UA	343.02	2.13	343.80	226.20	471.10	307.25	343.80	376.95
WBC	6.46	0.10	6.23	2.82	16.82	5.29	6.23	7.31
Plt	237.15	2.96	231.00	115.00	476.00	201.00	231.00	267.00
RBC	4.96	0.03	4.97	3.73	8.04	4.63	4.97	5.28
RDW	12.74	0.04	12.70	11.00	16.00	12.20	12.70	13.20
Hcy	15.06	0.83	11.80	5.90	114.20	9.00	11.80	15.83

The features with more missing values (> 40%) and unimportant can be deleted. Features with fewer missing values can be filled. We can use statistics to fill in mean, median, and mode. It is recommended to use the median for continuous values, excluding the influence of some large or small outliers. For discrete values, we can use mode to fill in.

### Feature construction

Based on the information obtained from data analysis and combined with the understanding of hypertensive renal damage, we analyze and construct the following features.

1. Personal information features: height, weight, age, sex, BMI, smoking or not, and body surface area(BSA).
2. Ambulatory blood pressure features: 24-h average SBP, 24-h average DBP, 24-h average heart rate, day average SBP, day average DBP, day average heart rate, night SBP drop rate, night DBP drop rate, blood pressure circadian rhythm, night average DBP, and night average SBP.
3. Blood biochemical and routine features: HDL, TG, FBG, UA, LDL, RDW, and BUN.

### Feature selection

Feature selection is also called feature subset selection or attribute selection. It refers to selecting a subset of features from all features to make the constructed model best [12]. In the application of data mining, the number of features is usually large, among which there may be uncorrelated features, and there may be interdependence between the features. It is easy to increase the model training time and cause a curse of dimensionality [13]. In addition, the model will also become complicated, and its generalization ability will decline.

For feature selection, we use the following methods:

1. Using the variance selection method, we calculated the variance of each feature and then eliminated the feature with variance more minor than the threshold.
2. We calculated the correlation coefficient and *P*-value between each feature and the target value using the correlation coefficient method.
3. Using the variance inflation factor to determine the correlation between variables to perform multicollinearity detection.
4. Using the random forest as the base model to train to get the importance of different features for selection.

### Model optimization and fusion

We use the K-Fold function for cross-validation in the scikit-learn(Python package) to divide the data into five sets of train sets and test sets to perform 5-fold cross-validation [14]. It can effectively avoid the risk of overfitting caused by limited data volume. The data distribution in the train set and test set is similar to the distribution of all data.

In order to determine the best prediction model, we use Random Forest, Extra-Trees, and XGBoost to train the data. During model training, we use grid search to adjust and optimize model parameters. Grid search is a model parameter optimization method [15] whose essence is an exhaustive method. We select a small finite set for each parameter to explore and carry out the Cartesian product on these parameters to obtain several sets of parameters. Then, grid search uses each set of parameters to train the model and picks out the best set of parameters [16].

**Fig. 2:**
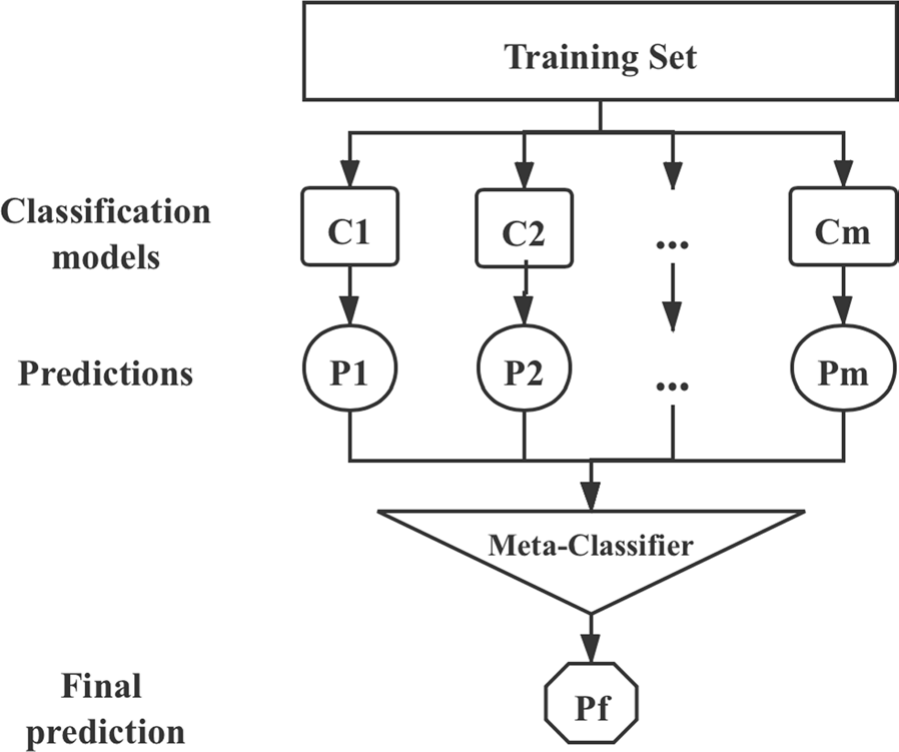
The structure of Stacking model fusion strategy

After the above model training is completed, we use the Stacking method to integrate the above models to build a new model to improve the prediction effect. The Stacking model fusion strategy is based on the idea of K-fold cross-validation, whose essence is a hierarchical model integration framework to stack the learning ability of different models for different features. However, as the number of layers increases, there is a risk of overfitting. Therefore, we usually use a two-layer model to reduce the number of data repeat training. The first layer model comprises several base learners whose input is the original train set. Moreover, the second layer model uses the output of the first layer model as the train set to retrain. The structure of the Stacking model fusion strategy is shown in Fig. 2.

We use the two-dimensional fusion model based on the stacking strategy. The first layer model uses the combination of random forest, extra trees, and XG-Boost as the base learner to train the data, and the second layer model uses XGBoost to train the output of the first layer model.

## Result

### Risk factors

**Fig. 3:**
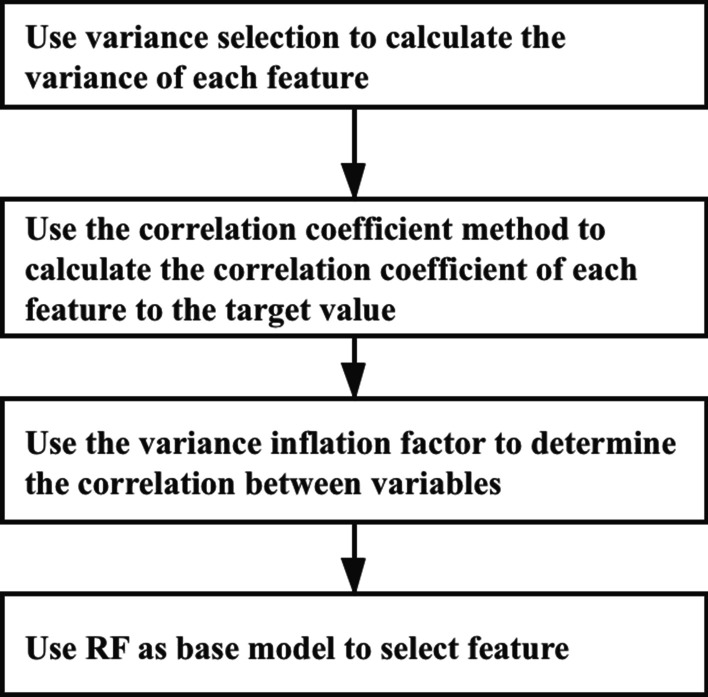
The steps of feature selection

The steps of feature selection are shown in Fig. 3. After feature selection, we select eight features. According to the order of importance from high to low, they are as follows: drop rate of systolic blood pressure at night(night SBP drop rate), red blood cell distribution width(RDW), blood pressure circadian rhythm, average diastolic blood pressure at daytime(day average DBP), body surface area(BSA), smoking, age, and HDL. The importance of features is shown in Table 6. Besides, we have compared full features with selected features results based on the Stacking strategy are shown in Table 7. It shows that the selected eight features are of great significance for predicting renal damage.

**Table 6:** The importance of features

Order	Feature name	Importance
1	Night SBP drop rate	0.39149
2	RDW	0.20044
3	Blood pressure circadian rhythm	0.15787
4	Day average DBP	0.07692
5	BSA	0.05067
6	Smoking	0.04294
7	Age	0.04236
8	HDL	0.03732

**Table 7:** The comparison of fivefold cross validation for full features vs selected features

Feature	Avg precision	Avg recall	Avg F1 score
Full features	0.89685	0.79086	0.82250
Selected features	0.93824	0.84595	0.88086

### Model

The precision, recall, F1 score results of 5-fold cross- validation for each model are shown in Table 8. In single model training, the effect of Random Forest and XGBoost is similar. Compared with Random Forest, Extra-Trees, and XGBoost, the two-dimensional fusion model based on the Stacking method has the highest precision, recall rate, F1 value. The first layer of the fusion model is consists of XGBoost, Extra-Trees, and RF. And the second layer of the fusion model is XG-Boost.

**Table 8:** The results of fivefold cross validation for each model

Model	Avg precision	Avg Recall	Avg F1 score
RF	0.92746	0.83227	0.86792
ExtraTrees	0.92378	0.80968	0.84462
XGBoost	0.93522	0.82510	0.86564
Stacking	0.93824	0.84595	0.88086

**Fig. 4:**
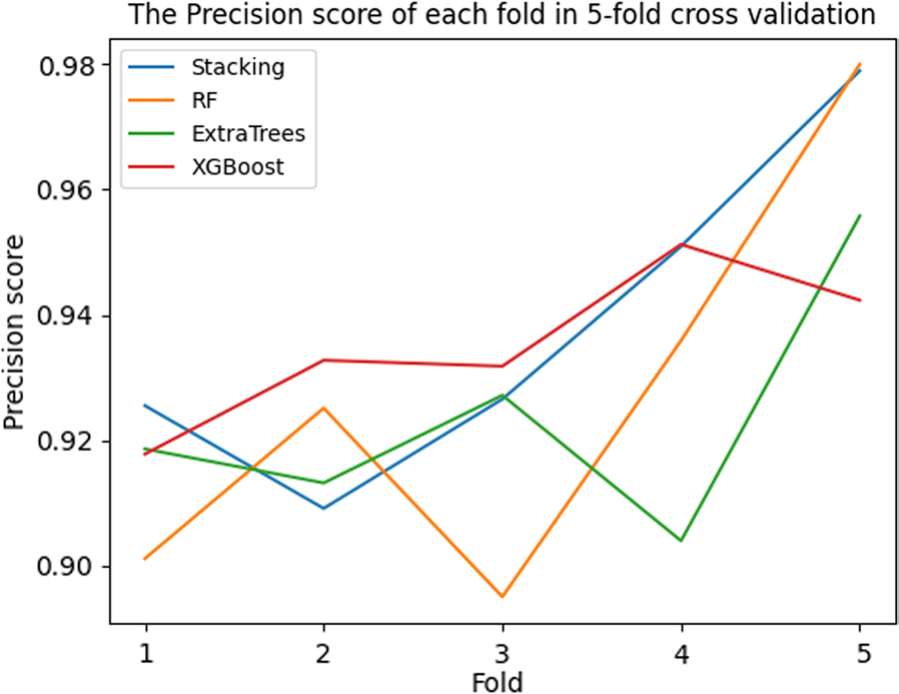
The precision of each fold

**Fig. 5:**
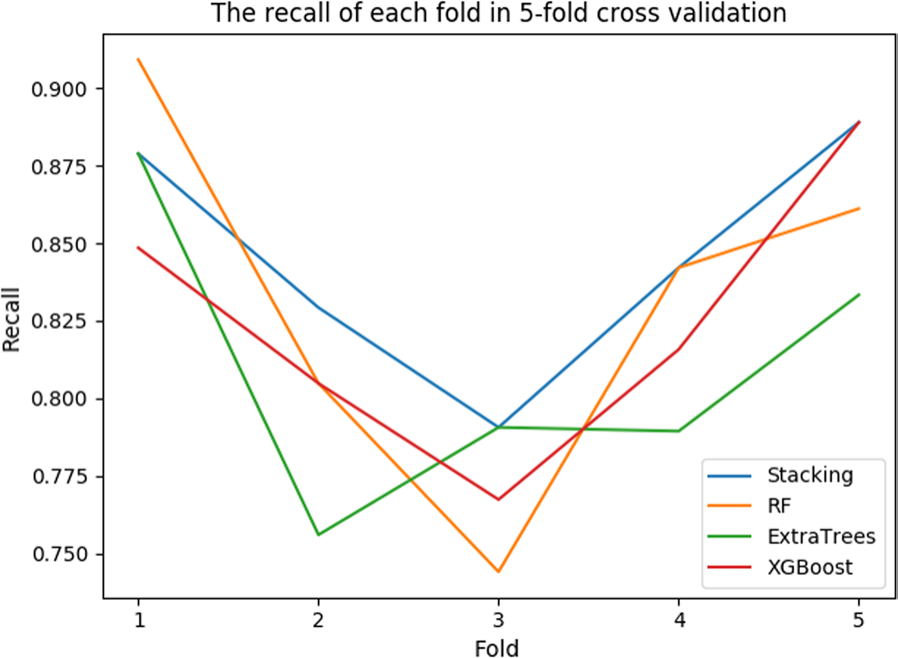
The recall of each fold

**Fig. 6:**
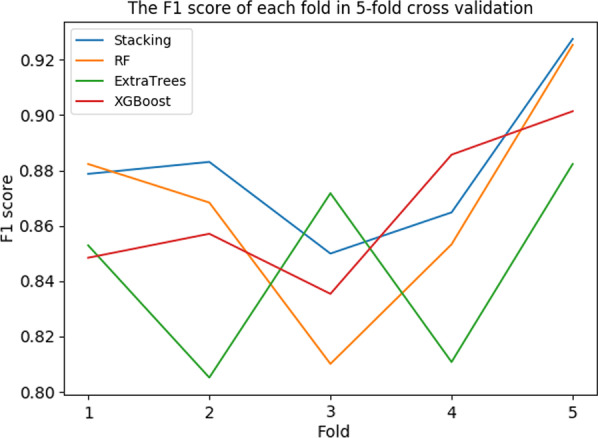
The F1 score of each fold

**Fig. 7:**
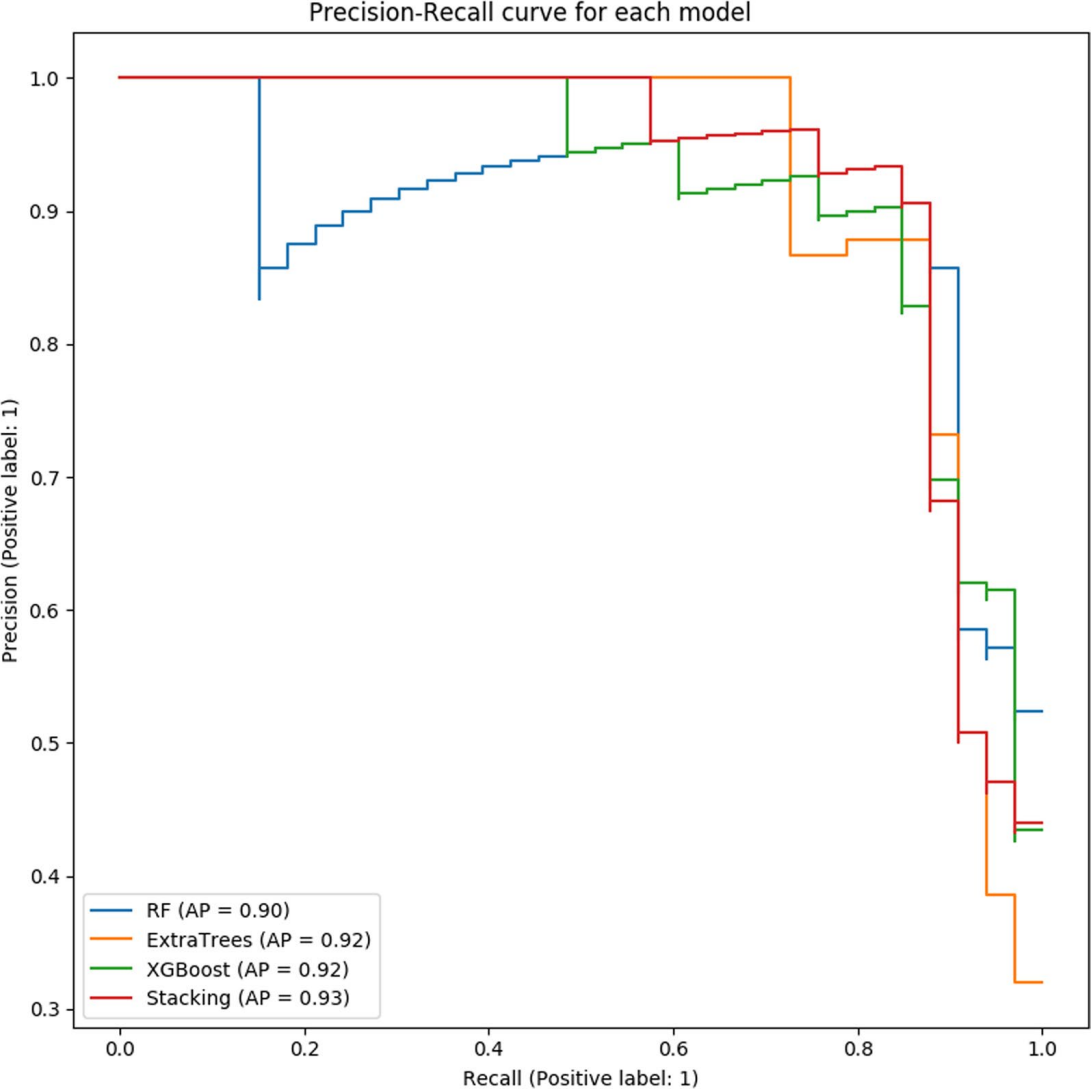
The Precision-Recall curve of each model

The precision of each fold in 5-fold cross-validation for each model is shown in Fig. 4. The recall is shown in Fig. 5. The F1 score is shown in Fig. 6. The Precision-Recall curve of each model is shown in Fig. 7. From the training results of each fold, we can find that the training effect of the fusion model based on the Stacking method in each fold is in the top two. It shows that the fusion model based on the Stacking method integrates the learning ability of different models for different features to improve the prediction effect on all data. In addition, as can be seen from Fig. 7, the stacking effect is the best of all models.

## Discussion

### Risk factor analysis

In screening CKD patients and monitoring renal function in the treatment, the main clinical index is serum creatinine. But serum creatinine assessment is not sensitive to detecting early subclinical changes and predicting renal function decline after treatment. In the preclinical stage of CKD, we need new monitoring indicators to evaluate such patients. Early renal damage can be judged by microalbuminuria and glomerular filtration rate (GFR). However, the role of urinary microalbumin has not been deemed significant due to red measurement errors. GFR is affected by many factors. Even though nuclear medicine method measurement is a gold standard, it is seldom carried out due to the complexity of cost and operation. The estimated GFR can not reflect the real renal function because the formula is complicated, and the results of different formulas are pretty different. This part of the study aims to understand the early renal damage of untreated hypertension patients, screen the relevant risk factors, and find out specific high-risk factors. It also provides quantitative indicators (early warning signals) for early renal damage hypertension patients and cardiovascular clinicians to prevent CKD’s progress better.

### Abnormal blood pressure indexes

The comparison results between the two groups show that the patients in the early renal damage hypertension group are older than those in the control group. Moreover, their HDL and BSA are lower, and their blood pressure index is higher than the control group. Especially the nighttime blood pressure level and blood pressure variability. Further analysis shows that abnormal blood pressure rhythm in the two groups is quite different. In the early renal damage group, the proportion of non-dipper type, reverse-dipper type, and deep dipper type account for 75.9%, 14.1%, and 3.1%, respectively, while the normal rhythm is more petite than 10%. In contrast, 72.4% of the patients in the control group have normal blood pressure rhythm. The blood pressure circadian rhythm analysis indicates that the difference in nighttime blood pressure drop rate between the two groups is statistically significant. The drop of nighttime blood pressure is weakened in the early renal damage group.

Cheng Dong et al. [17] found that the drop of blood pressure at night was a significant predictor of renal damage in hypertensive patients. Mingling et al. [18] studied the albuminuria and blood pressure level of hypertension patients in five different regions in China. It found that poor blood pressure control was an essential factor for proteinuria. Effective blood pressure control was critical in reducing proteinuria, improving endothelial function, and renal protection. Our study finds that SBP, DBP, and PP(clinic, 24-h, day, night) in the ACR positive group are higher than those in the control group (*P* < 0.05) to indicate that the higher the blood pressure level, the higher the incidence of ACR. In addition, our study also concludes that the drop rate of nighttime systolic blood pressure and the average diastolic blood pressure in the daytime are risk factors for ACR occurrence. Furthermore, it shows that controlling blood pressure levels is significant for patients with hypertension.

### Abnormal blood pressure rhythm

People’s blood pressure is higher in the day and lower at night. That is to say, the blood pressure drops during sleep at night and is the lowest in the early morning; the blood pressure starts to rise in the early morning and then presents the first peak. In normal people and patients with arytenoid rhythm hypertension, sympathetic activity, cardiac output, and blood pressure decrease during sleep. Huijuan et al. [19] found that compared to patients with dipper hypertension, patients with non-dipper and anti-dipper hypertension were closely related to early renal damage indicators. It indicated a close relationship between the abnormal circadian rhythm of blood pressure and early renal damage. Zeming et al. [20] found that the abnormal blood pressure circadian rhythm was the important factor causing the early-stage renal damage, reverse-dipper make early-stage renal damage was more significant than in the control group. Nighttime systolic blood pressure levels and blood pressure circadian rhythm had crucial clinical significance for earlystage renal damage in patients with hypertension.

This study suggests that the ambulatory blood pressure level of patients with early renal damage of hypertension increases with the increase of urinary microalbumin, which is manifested by the increase of nighttime blood pressure, significantly the increase of nighttime diastolic blood pressure. The study also finds that the early renal damage of hypertension is often accompanied by abnormal blood pressure circadian rhythm, and it has existed in hypertensive patients without microalbuminuria.

In comparison with the control group, the patients with dipper and non-dipper rhythm, the patients with anti-dipper rhythm have higher ACR, night SBP, night DBP, and night PP. While the decrease of eGFR is more prominent than the control group. It suggests that anti-dipper rhythm plays a relevant and independent role in the occurrence and development of early renal damage in hypertension, regardless of whether the clinic blood pressure level and dynamic blood pressure level are the same. In addition, nighttime blood pressure level and circadian rhythm are positively correlated with ACR but not with eGFR. It suggests that the anti-dipper blood pressure circadian rhythm is independently correlated with microalbuminuria in patients with hypertension. Our study finds that all patients in the anti-dipper rhythm group have early renal damage, which may be due to a small sample size or a biased selection. However, it is enough to show that the early renal damage in the anti-dipper rhythm group is more severe than in the control group. In the future, we need larger samples and more evidence to confirm the causal relationship between the anti-dipper rhythm and early hypertensive renal damage.

### Red blood cell distribution width

A series of studies confirmed the correlation between RDW(Red cell Distribution Width) and hypertension. Tanindi et al. [21] found that hypertensive patients had higher RDW levels and higher systolic and diastolic blood pressure than prehypertensive patients. Perlstein et al. also found that the systolic blood pressure level and the proportion of hypertensive patients were significantly increased in people with higher RDW [22]; Formal et al. found that RDW is closely related to the delay in the reduction of the nighttime blood pressure in hypertensive patients, which is an independent predictor of nighttime non-dipper blood pressure [23]. Correlation between RDW and renal function has also been reported. Ujszaszi et al. [24] observed that RDW was independently associated with decreased renal function in renal transplant patients and considered it as a potential new auxiliary parameter for clinical evaluation for patients with chronic kidney disease. Recently, Solak et al. found that RDW was significantly increased in patients with CKD from stage 1 to stage 5, which was closely related to endothelial dys- function in patients with chronic kidney disease [25]. However, the above studies are limited to the CKD population, and their results may be affected by drug and disease progression.

This study finds that RDW is associated with early renal damage in hypertensive patients, and the ACR ratio also tends to increase as RDW increases. Combined with the data in this group, hypertensive patients have different degrees of early renal damage. RDW is a sensitive indicator for the diagnosis of early renal damage in hypertensive patients, and RDW is a common item of routine blood examination. The method is convenient, fast, and inexpensive. Of course, RDW, as an indicator of risk assessment of early renal damage in hypertensive patients, still needs evidence support from prospective studies in the future.

### Model analysis

When we use the Stacking method for model fusion, the corresponding results may be different when the model combination of each layer is different. In order to determine the best combination of models, the first layer model uses the random combination of Random Forest, Extra-Trees, and XGBoost as the basic learner, and the second layer uses XGBoost(from the figure of precision and recall, the RF is unstable and the generalization ability is weak, so XGBoost is used). Then, we carry out 5-fold cross-validation on the data. Through comparison, we can find that the average precision of two-dimensional fusion model based on Random Forest is the best. However, the random combination of Random Forest, Extra-Trees, and XG- Boost is the most stable. And the F1 and recall of two- dimensional fusion model based on XGBoost, Random Forest and Extra-Trees is the best. Therefore, the random combination of Random Forest, Extra-Trees, and XGBoost is the best. The results of 5-fold cross-validation for each model combination are shown in Table 9.

**Table 9:** The results of fivefold cross validation for each combination. ET is ExtraTrees, RF is Random Forest, and XGB is XGBoost

First layer	Avg precision	Avg recall	Avg F1 score
RF	0.94490	0.82599	0.85942
ET	0.94127	0.81607	0.84857
XGB	0.93829	0.82510	0.86072
RF + ET	0.93048	0.82050	0.85103
XGB + RF	0.92910	0.82510	0.86564
XGB + ET	0.93565	0.82510	0.86564
XGB + ET + RF	0.93824	0.84595	0.88086

## Limitations

There are some limitations in current research. In the aspect of screening risk factors of renal damage in hypertension, due to the inherent limitations of a case-control study, to further clarify the relationship between the above risk factors and early renal damage in hypertensive patients, it needs to be further confirmed by more centers, larger samples, and prospective studies. In establishing an early warning model of renal damage, a small sample is a severe limitation, which will affect the precision and generalization ability of the model. However, the small sample and data imbalance are common in clinical research. How to apply the model to clinical research still needs further exploration.

In order to overcome the limitations of this study, we should collect more data about hypertensive patients with early renal damage to validate and optimize the model. Moreover, we may solve small sample limitations by few-shot learning. In addition, we could fuse other models with better effects to get the better result [26].

## Conclusion

This study mainly carries out the application research of data mining combined with routine clinical items in early warning of renal damage in hypertensive patients. We then use feature engineering and risk factor analysis to screen for risk factors such as the drop rate of systolic blood pressure at night, red blood cell distribution width, blood pressure circadian rhythm, and the average diastolic blood pressure at daytime as early renal damage’s warning sign. On this basis, the early-warning model of early kidney damage constructed by the Stacking model fusion strategy has a better effect than the single model. This model can diagnose renal damage in hypertensive patients and has important significance for screening high-risk populations. We can try to fuse the better model and test its prediction effect in the future. At the same time, the methods and ideas of this research can also provide new methodological references for similar early-warning research and evaluation.

## Data Availability

The datasets used and/or analysed during the current study are available from the corresponding author on reasonable request.

## Abbreviations

BMI: Body mass index
BSA: Body surface area
FBG: Fasting blood-glucose
2hPBG: 2H postprandial blood glucose
TG: Triglyceride
HDL-C: High density lipoprotein cholesterol
LDL-C: Low density lipoprotein cholesterol
BUN: Blood urea nitrogen
Scr: Serum creatinine
UA: Uric acid
eGFR: Estimated glomerular filtration rate
ACR: Albumin-to-creatinine ratio
hs-CRP: High-sensitivity C-reactive protein
RDW: Red cell distribution width
OBPM: Office blood pressure measure
ABPM: Ambulatory blood pressure monitoring
SBP: Systolic blood pressure
DBP: Diastolic blood pressure
PP: Pulse pressure
CV: Coefficient of variability
CKD: Chronic kidney disease
LAD: Left atrium diameter
LVEDD: Left ventricular end diastolic diameter
LVRWT: Left ventricular relative wall thickness
MLVRWT: Maximal left ventricular relative wall thickness
LVMI: Left ventricular mass index
RF: Random forest
XGB: XGBoost
ET: Extra trees
